# Stepwise approach in the management of penile strangulation and penile preservation: 15-year experience in a tertiary care hospital

**DOI:** 10.1080/2090598X.2019.1647677

**Published:** 2019-08-21

**Authors:** Sandeep Puvvada, Priyatham Kasaraneni, Ramesh Desi Gowda, Prasad Mylarappa, Manasa T, Kanishk Dokania, Abhishek Kulkarni, Vivek Jayakumar

**Affiliations:** Department of Urology, MS Ramaiah Medical College, Bengaluru, India

**Keywords:** Penile strangulation, penile asphyxia, metallic ring, penile incarceration, penile injury, cutting device, string technique, penile preservation

## Abstract

**Objective**: To present our stepwise approach to the management of penile strangulation and penile preservation with 15 years’ experience in a tertiary care hospital, as penile strangulation is a rare urological emergency that requires immediate attention.

**Patients and methods**: A prospective observational study was performed from March 2003 to December 2018 of patients presenting with penile strangulation to our hospital.

**Results**: Nine patients with penile strangulation presented to us between March 2003 and December 2018. The most common motive for the application of a foreign body was sexual gratification (four patients). Three of the nine patients had a mental disorder. Objects used for strangulation included: metallic nut (three), metallic ring (two), plastic bottle (two), wooden hole (one), hammer head (one), and horse hair to control bleeding during circumcision (one). Most of the foreign bodies were located in the proximal penile region. The mean operative time was 38 min and three of the nine patients had complications.

**Conclusions**: Penile strangulation is one of the rare urological emergencies experienced by a urologist. Removal of the foreign body can be difficult and there is no universal method of removal, as each case differs. So, following our stepwise approach can aid in removal of foreign body quickly and preserve the penis from fatal outcomes. Urologist should be aware of all the available armamentarium used for the removal of such foreign bodies.

**Abbreviation**: SPC: suprapubic cystostomy

## Introduction

Penile strangulation by a foreign body is a rare condition and only few case series have been published, with <100 case reports. It is an uncommon urological emergency, if not treated as soon as possible it can lead to complications such as gangrene and amputation of the penis [1]. There is no standard of care that has been found to be superior, with each case managed individually according to its clinical findings and operative settings [2]. Gauthier reported the first case of penile strangulation in literature in 1755 [3]. The most common cause of penile strangulation is by a foreign body that compresses the penis circumferentially, and include objects made of metallic or non-metallic materials. Thin non-metallic constrictive objects are easy to remove. The various objects causing penile strangulation published in the literature include: heavy metal rings, hammer head, plastic bottle necks, sprockets, and plumbing cuffs [4]. Metal objects are very difficult to remove and cutting them is the most common procedure to remove them [4,5]. But most hospitals are not equipped with appropriate cutting tools, and urologists are not aware of the equipment used to cut them. Furthermore, cutting the metallic object is a time consuming process [4]. So, the urologist should be ready and aware of the equipment required for cutting such foreign bodies as quickly as possible to manage the medical emergency. So, the aim of our present study was to frame a stepwise approach to managing penile strangulation quicker to prevent complications such as gangrene and amputation.

## Patients and methods

A prospective observational study was performed from March 2003 to December 2018 on patients presenting to our hospital with penile strangulation. Patient’s clinical history, physical examination findings, and duration of strangulation were documented; there is no necessity for any diagnostic test as it is clinically evident the type of foreign body used. Grading of the injury was done as per the Bhat et al. [5] grading system:

1. Grade 1, oedema of distal penis. No evidence of skin ulceration or urethral injury.
2. Grade 2, injury to skin and constriction of corpus spongiosum, but no evidence of urethral injury. Distal penile oedema with decreased penile sensation.
3. Grade 3, injury to skin and urethra but no urethral fistula. Loss of distal penile sensations.
4. Grade 4, complete division of corpus spongiosum leading to urethral fistula and constriction of corpora cavernosa, with loss of distal penile sensations.
5. Grade 5, gangrene, necrosis, or complete amputation of the distal penis.

**Figure 1.**
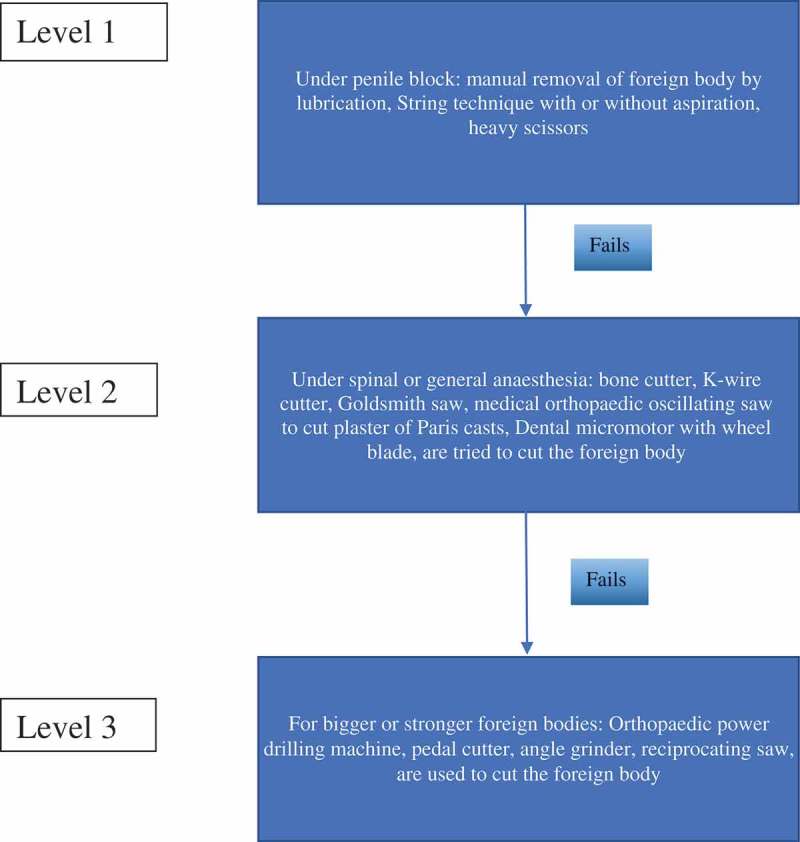
Stepwise approach.

Management was via a stepwise approach followed by our department as shown in Figure 1.

As soon as the patient presents to the hospital, we assess the patient vitals to assess whether he is haemodynamically stable, followed by a clinical history, examination of genitals, and grading of the injury. We give analgesics, third-generation cephalosporin antibiotics, and tetanus prophylaxis before any intervention. Then the above-mentioned armamentarium (Figure 1) available on the hospital premises were made ready and treatment given as per the flowchart in Figure 1.

As the first step (Level 1), we use a penile block and try to remove the foreign body by manual removal after lubrication with xylocaine jelly/removal using the string technique with or without aspiration of corporal blood by passing an 18-G needle to facilitate removal/cutting of non-metallic foreign bodies with heavy scissors.

If Level 1 fails, we then transfer the patient to the operating room and under spinal or general anaesthesia start Level 2 techniques. The reason for spinal or general anaesthesia is that patient immobility is required for precision and to permit removal of the foreign body without injury to the surrounding structures. The various instruments used in Level 2 include: bone cutter, K-wire cutter, Goldsmith saw, medical orthopaedic oscillating saw, and dental micromotor with wheel blade to cut plaster of Paris casts. One of these instruments is selected and used to try to cut through the foreign body.

**Figure 2.**
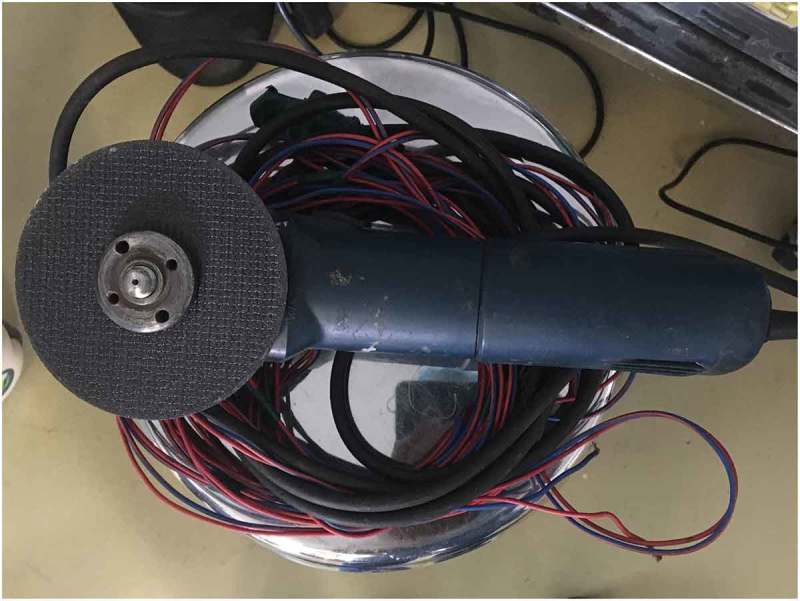
Angle grinder used for cutting foreign body.

If Level 2 fails, then more powerful devices (Level 3) such as an orthopaedic power drilling machine, pedal cutter, angle grinder (Figure 2), and reciprocating saw, are used to cut the foreign body.

**Figure 3.**
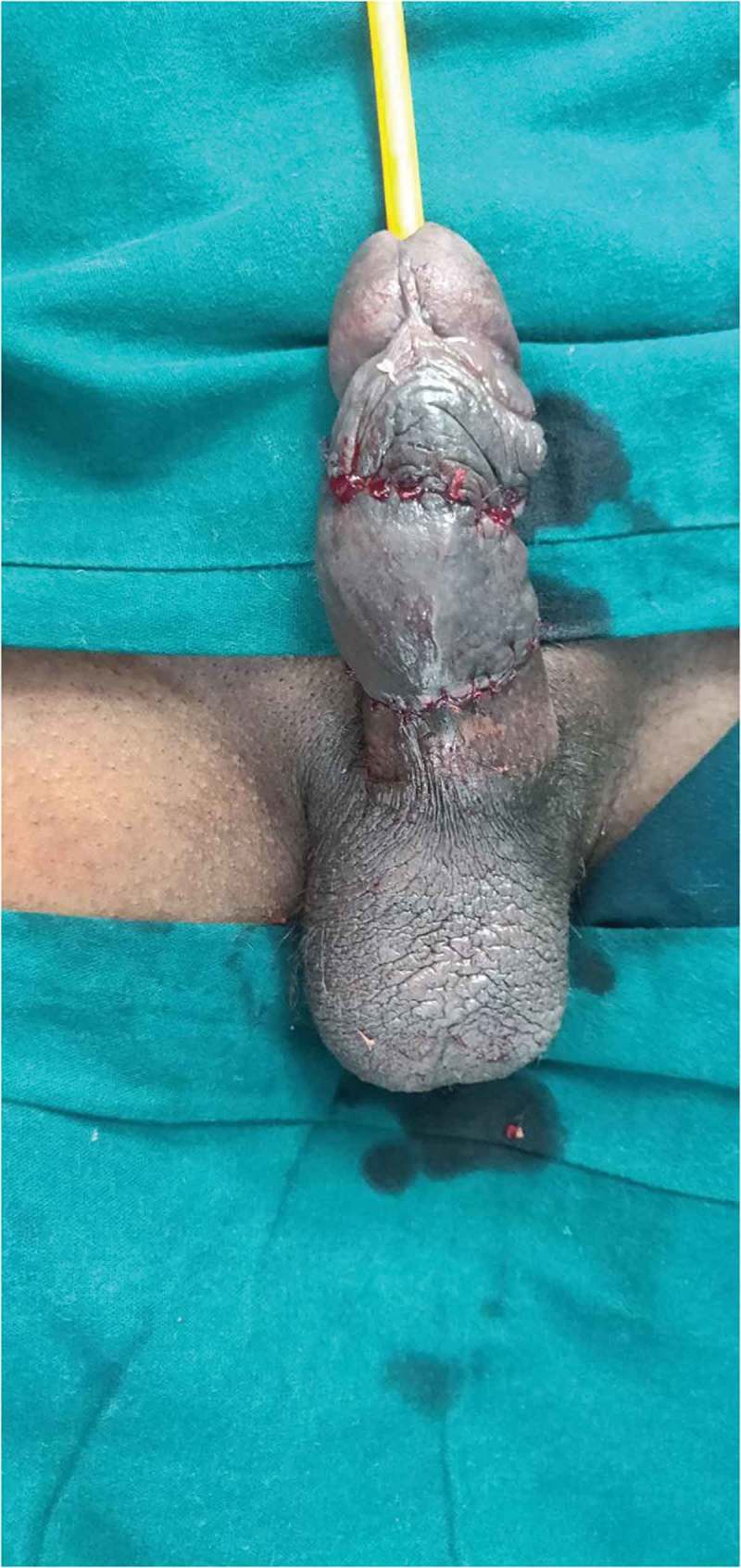
Post debridement, delayed closure.

After removal of the foreign body, we visually assess for any urethral injury and pass a 16-F Foley catheter, which is left *in situ* for 2 days. If catheterisation is not possible, then a suprapubic cystostomy (SPC) is performed and the patient is re-assessed after 3–6 weeks by retrograde urethrography and managed accordingly. Postoperative Doppler ultrasonography of the penis is done within 12 h after removal of the foreign body in all the patients. The skin of the penis is examined and debridement is done if the tissue is not viable with delayed closure (4–6 weeks) if the wound is not healthy (Figure 3). Skin grafting was done when required. The antibiotics were continued for 5 days and anti-oedematous agents, such as trypsin and chymotrypsin, were given for 7 days. The patient was followed-up on postoperative days 7 and 30 with penile Doppler ultrasonography. Patients with unhealthy wounds are re-assessed every week.

## Results

Table 1 gives a brief overview of our results.

**Table 1.** Details of the nine cases.

Patient number	Age, years	Motive	Applied by	Sexual Life	Mental status	Object	Grade of injury	Duration of injury	Mode of removal	Remarks
1	65	Suicide	Self	Married	Mental disorder	Plastic bottle + metallic nut	3	15 days	Angle grinder	
2	26	Sexual gratification	Self	Unmarried	Mental disorder	Hammer head	1	6 h	String technique with aspiration	
3	1	Circumcision – hair tourniquet syndrome	Medically unqualified individual	Unmarried	Normal	Horse hair	4	4 h	Surgical scissors	Urethral reconstruction done
4	62	Assault	Others	Married	Normal	Metallic nut	5	2 months	Auto amputation of penis	SPC, debridement and delayed closure, perineal urethrostomy
5	34	Enhance erection	Self	Married	Normal	Plastic bottle	1	3 h	Heavy scissors	
6	28	Sexual gratification	Self	Married	Normal	Metallic Ring	2	4 h	Bone cutter	
7	22	Sexual gratification	Self	Unmarried	Mental disorder	Wooden hole	3	3 days	Medical orthopaedic oscillating saw to cut plaster of Paris casts	SPC done, lost to follow-up, presented with urethro–cutaneous fistula
8	48	Enhance erection	Self	Married	Normal	Metallic Ring	3	2 h	Pedal cutter	SPC done, skin grafting needed at follow up
9	52	Sexual gratification	Self	Married	Normal	Metallic Nut	1	8 h	Angle grinder	

Nine patients with penile strangulation presented to us between March 2003 and December 2018. The youngest patient was aged 1 year and the oldest was 62 years. The most common motive for application of the foreign body was sexual gratification (four patients), followed by to enhance erection (two), suicide (one), hair tourniquet syndrome (one), and assault (one).

**Figure 4.**
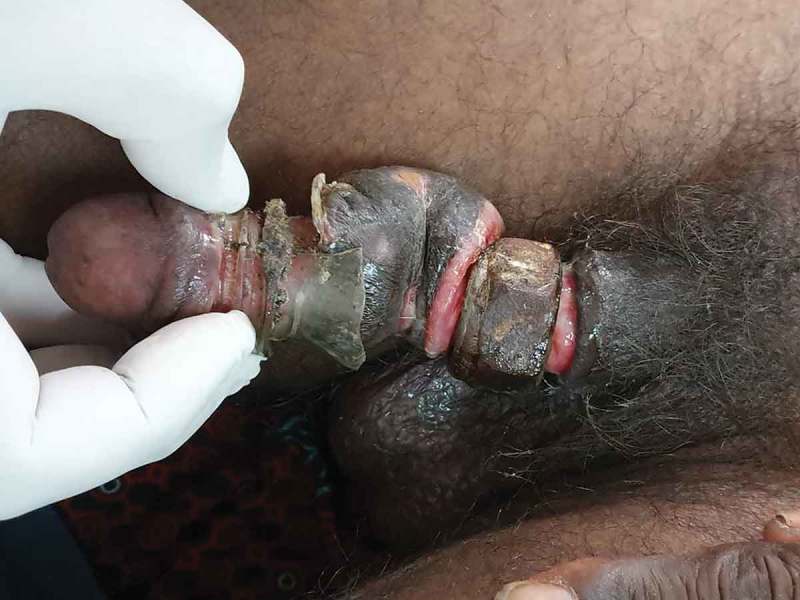
Penile strangulation by two objects; a plastic bottle and metallic nut.

**Figure 5.**
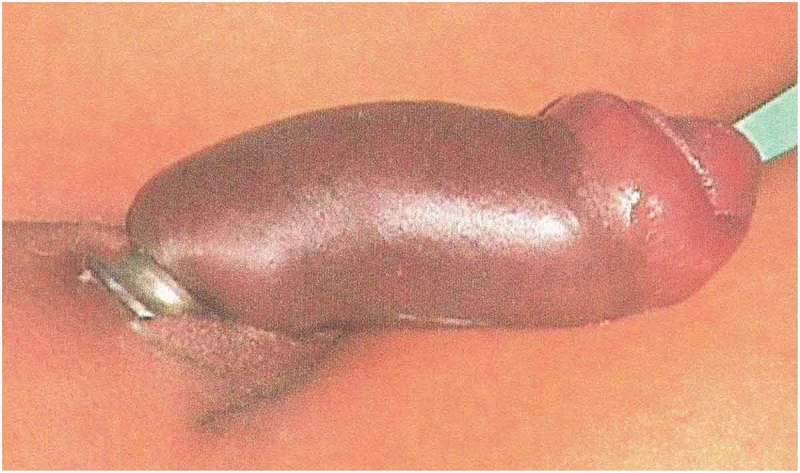
Metallic ring at the proximal penis with distal penile oedema and bluish discolouration of skin.

Six patients were married and three (one child) were unmarried. Three of the nine patients had a mental disorder. One patient used two foreign bodies, whereas rest used only one. The objects used for strangulation were: metallic nut (three patients; Figure 4), metallic ring (two; Figure 5), plastic bottle (two; Figure 4), wooden hole (one), hammer head (one; Figure 6(a,b)), and horse hair to control bleeding during circumcision (one).

**Figure 6.**
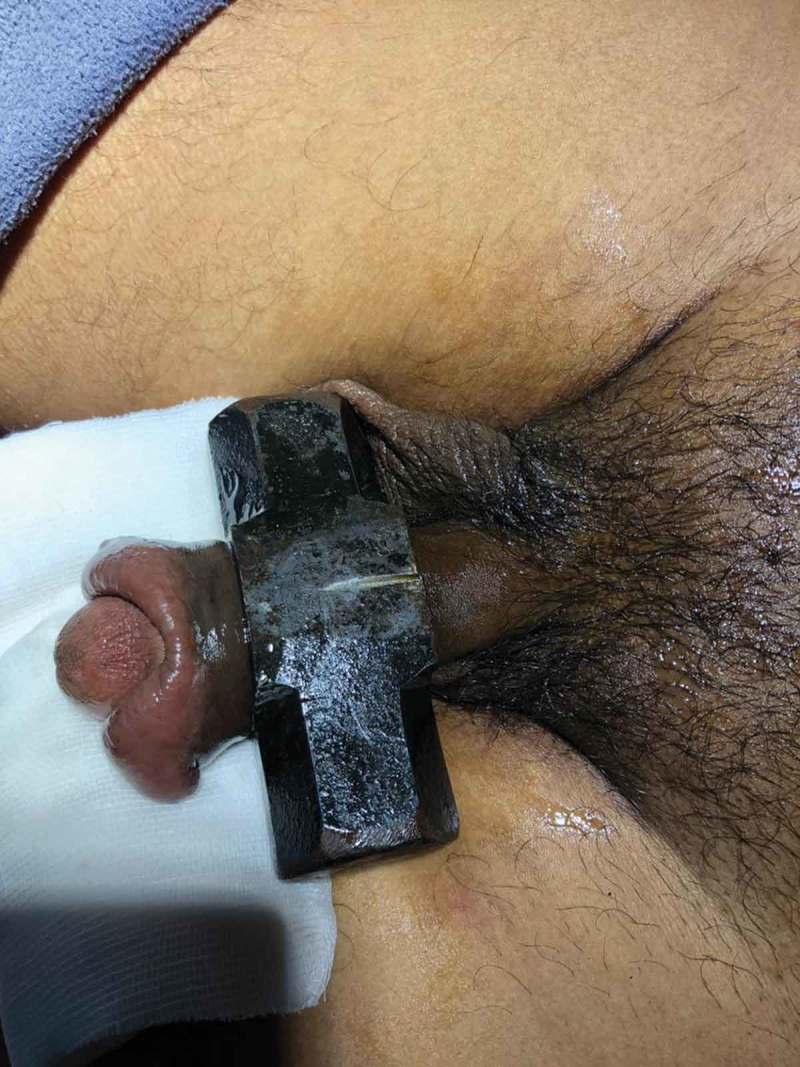
(a) Penile strangulation by hammer head in mid penile region with distal penile oedema.

**Figure 6.**
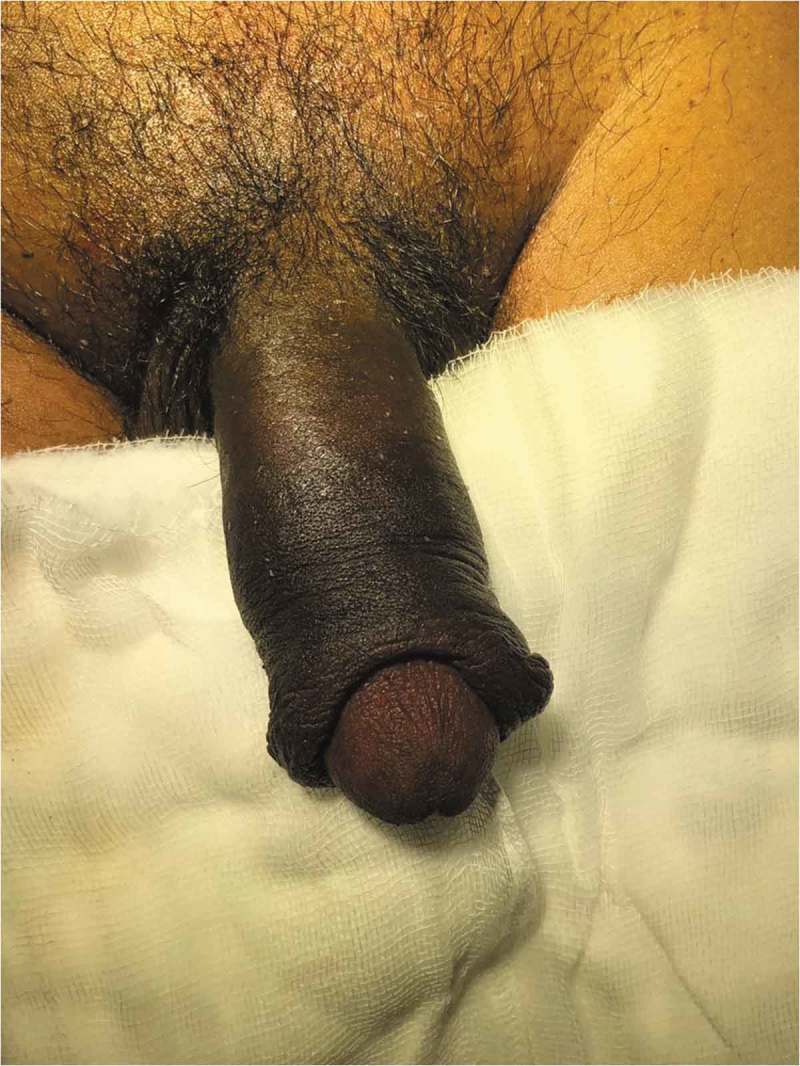
(b) After removal of hammer head foreign body by string technique with aspiration.

Seven of the nine patients presented with Grade ≤3 and two with Grade ≥4 injuries. The most common presentation was penile oedema and one patient presented with auto-amputation of penis due to penile strangulation by a metallic nut (Table 2).

**Table 2.** Incidence of presenting complaints.

Presenting complaint	*n/N*
Penile oedema	8/9
Urinary retention	5/9
Decreased penile sensation	4/9
Ulceration	4/9
Auto-amputation	1/9

Most of the foreign bodies were located in the proximal penile region (four of nine), one was distal and three were mid-penile. The mean operative time was 38 min.

**Figure 7.**
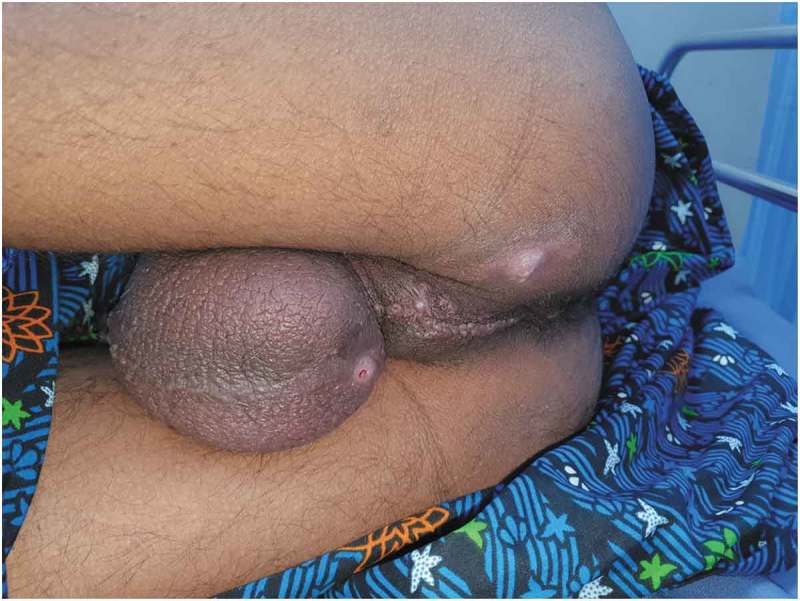
Urethro–cutaneous fistula.

The complications are described in Table 3. For three patients, SPC was needed as per urethral catheterisation was not possible. One patient lost to follow-up for 1 year had his SPC removed in a local hospital, he later presented back to us with urethro–cutaneous fistula (Figure 7) secondary to urethral stricture, SPC was repeated and after 3 months we excised the fistulous tract and he underwent anastomotic urethroplasty. The other patient had short segment stricture of the proximal penile urethra and he underwent visual internal urethrotomy. This patient is on regular follow-up with uroflowmetry. One patient presented with auto-amputation of penis with an infected wound over the detached penile site, so SPC was performed as a diversion procedure to allow healing followed by perineal urethrostomy after 1 month.

**Table 3.** Complications.

Complication	*n/N*
Stricture	2/9
Urethro–cutaneous fistula	1/9
Wound infection	1/9

One patient developed wound infection for which debridement and skin grafting was needed when the wound was healthy.

## Discussion

Cases of penile strangulation have been published for all age groups, with the foreign body used being placed most frequently for sexual gratification or by psychiatric patients. The foreign body is usually inserted on a flaccid penis and it becomes irremovable after erection. Later, due to oedema, it strangulates the genitalia. Prolonged constriction can lead to penile compartment syndrome, with an initial obstruction of both venous and lymphatic outflow distal to the device followed by arterial inflow obstruction, ultimately leading to tissue ischaemia and necrosis [6,7]. Penile strangulation is a urological emergency that should be promptly treated to decompress the involved tissues. Various techniques have been described in the literature; however, there is no universal technique to deal with the variety foreign bodies.

**Figure 8.**
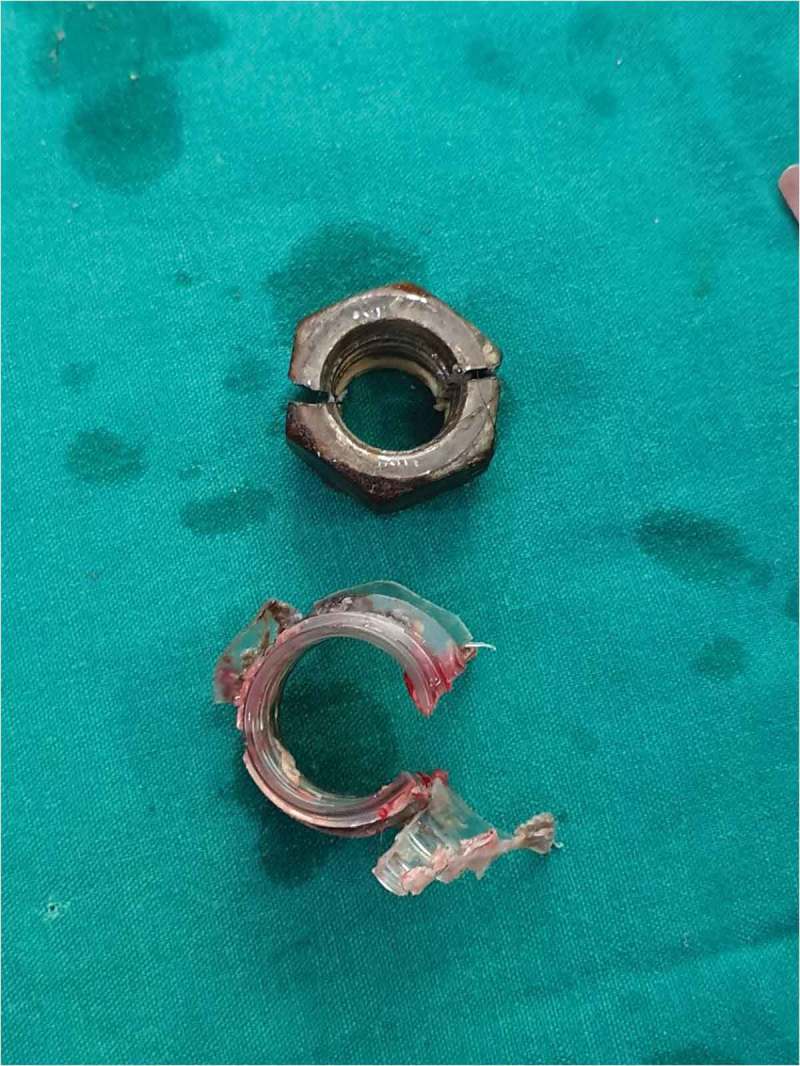
Metallic objects should be cut at two sites to facilitate easy removal whereas plastic bottle can be cut at one site.

Most of the case reports have a single constricting foreign body, whereas we had a patient with penile strangulation with two foreign bodies (Figure 8). However, a case report of penile strangulation with seven rings has been reported [8]. The most common motive for penile strangulation in our present study was sexual gratification (four of nine patients) followed by desire to enhance erection, assault, and hair tourniquet syndrome. The two patients who used a foreign body to enhance their erection had a history of erectile dysfunction. A 1-year-old patient had penile strangulation due to hair tourniquet syndrome, as a medically unqualified individual applied a horse hair to control bleeding at the post-circumcision site. Hair tourniquet syndrome has been published for penile hair [9,10], but penile strangulation from the application of horse hair has not been reported before. Penile strangulation has even been reported in literature in Parkinson’s disease [11]. Three of our present patients had a mental disorder, so after treatment of their penile strangulation, they were referred to a psychiatrist for psychotherapy.

The purpose of formulating a stepwise approach is to deal with the removal of a foreign body faster. In the present study our mean operative time was 38 min, whereas Shukla et al. [12] had an operative time range of 30–100 min.

**Figure 9.**
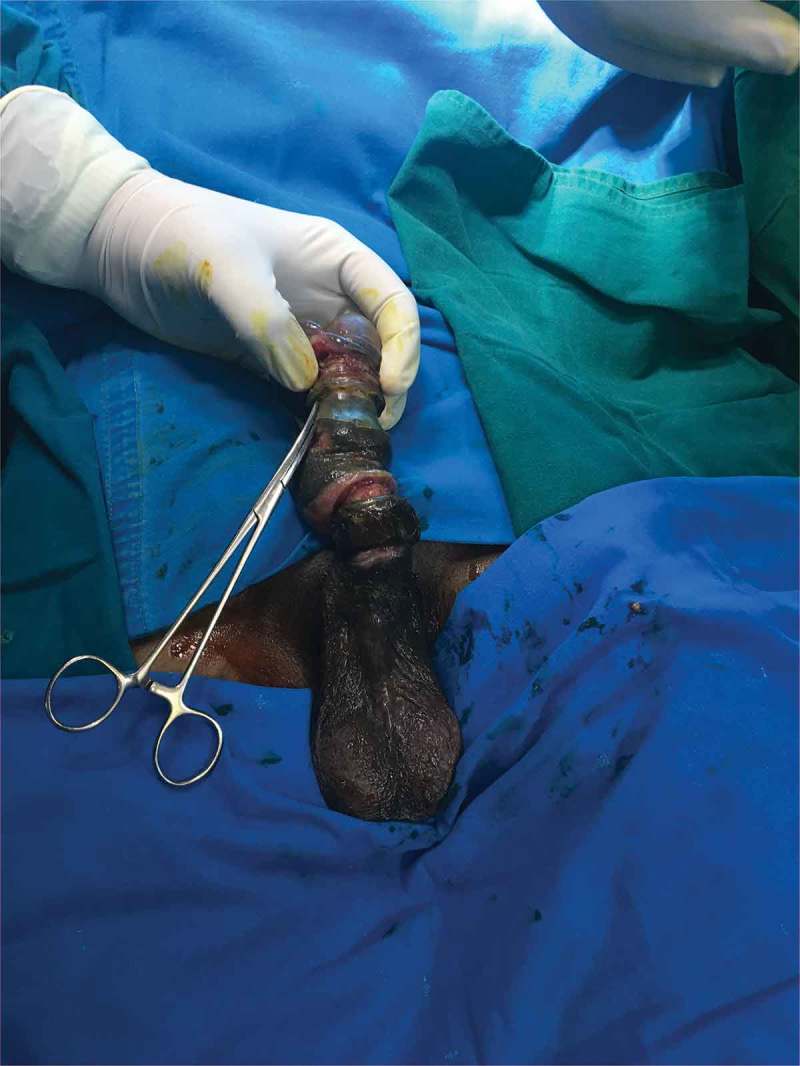
Trying Level 1 technique – string method.

**Figure 10.**
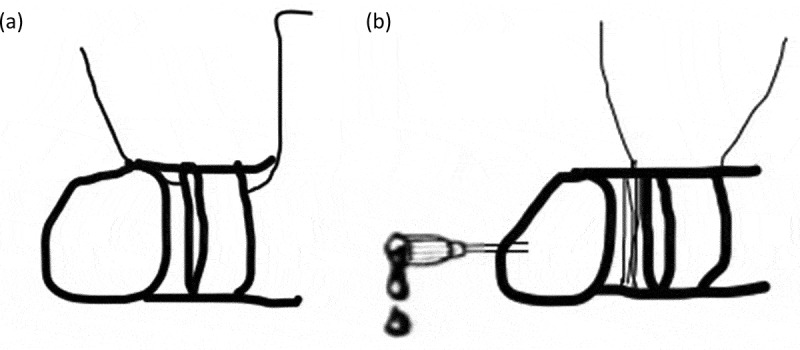
(a) Removal of foreign body by string technique. (b) Removal of foreign body by string technique combined with aspiration of corporal blood.

Level 1 techniques (Figure 9), under penile block, can be used for patients who present early, and even metallic foreign bodies can be removed with this technique. We tried removal with a modified string method in a patient (Figure 10(a,b)), this technique was published by Noh et al [3], and the purpose of corporal aspiration using an 18-G needle is to reduce the swelling and ischaemia to facilitate removal. Removal of metal objects causing penile strangulation using a silk winding method without aspiration has been reported by Dong et al. [13]. Non-metallic foreign bodies that are thin can be removed using heavy scissors, as we did for a plastic bottle top. Level 1 techniques can be performed bedside.

**Figure 11.**
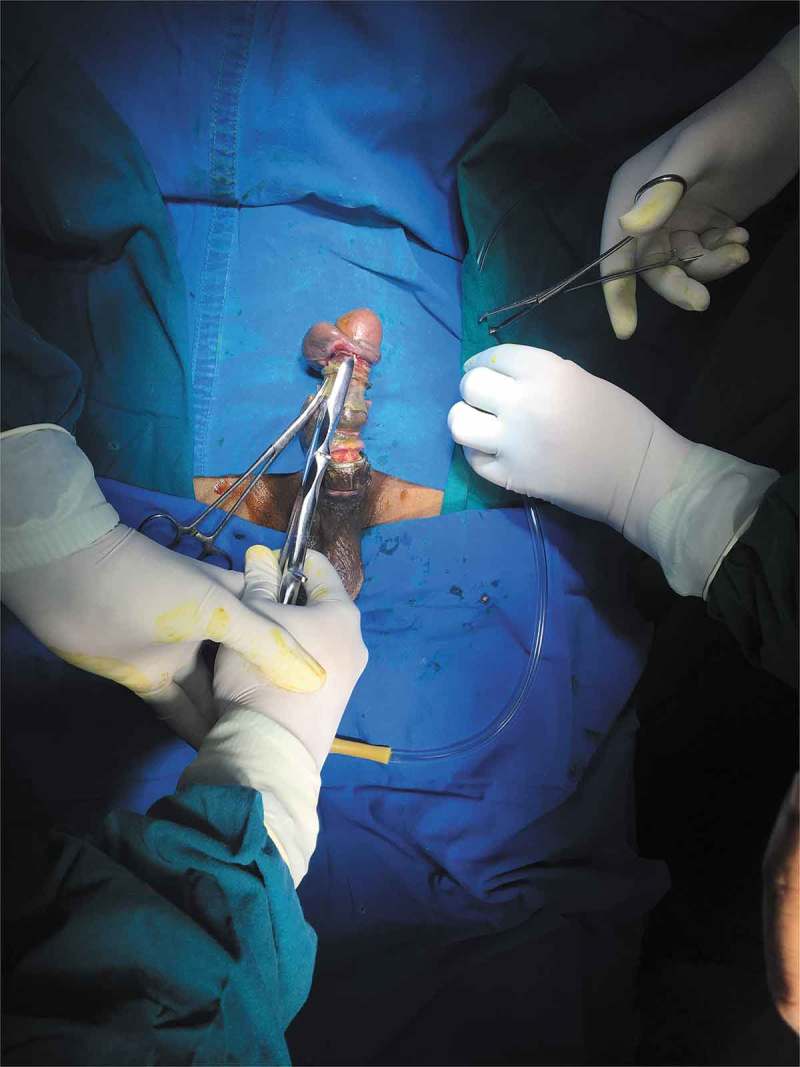
Trying Level 2 device – bone cutter.

If Level 1 fails then we switch to Level 2 (Figure 11). Level 2 is usually needed for thick non-metallic objects or thin metallic objects. The patient should be under spinal or general anaesthesia to immobilise the patient (as the patients will be in a state of panic and will be more frightened by the instrument sounds during the removal of the foreign body) and prevent injury to the surrounding structures whilst removing the foreign body. The different instruments used include: bone cutter, K-wire cutter, Goldsmith saw, medical orthopaedic oscillating saw to cut plaster of Paris casts, and dental micromotor with wheel blade. We used a bone cutter for one case and a medical orthopaedic oscillating saw to cut plaster of Paris casts for another case. Sawant et al. [14] used a K-wire cutter, Abd El Salam et al. [15] used a bone cutter, and Paonam et al. [16] used a micromotor wheel-shaped bur to cut through metal rings. Whilst, May et al. [17] used an oscillating splint saw to cut through a plastic bottle.

**Figure 12.**
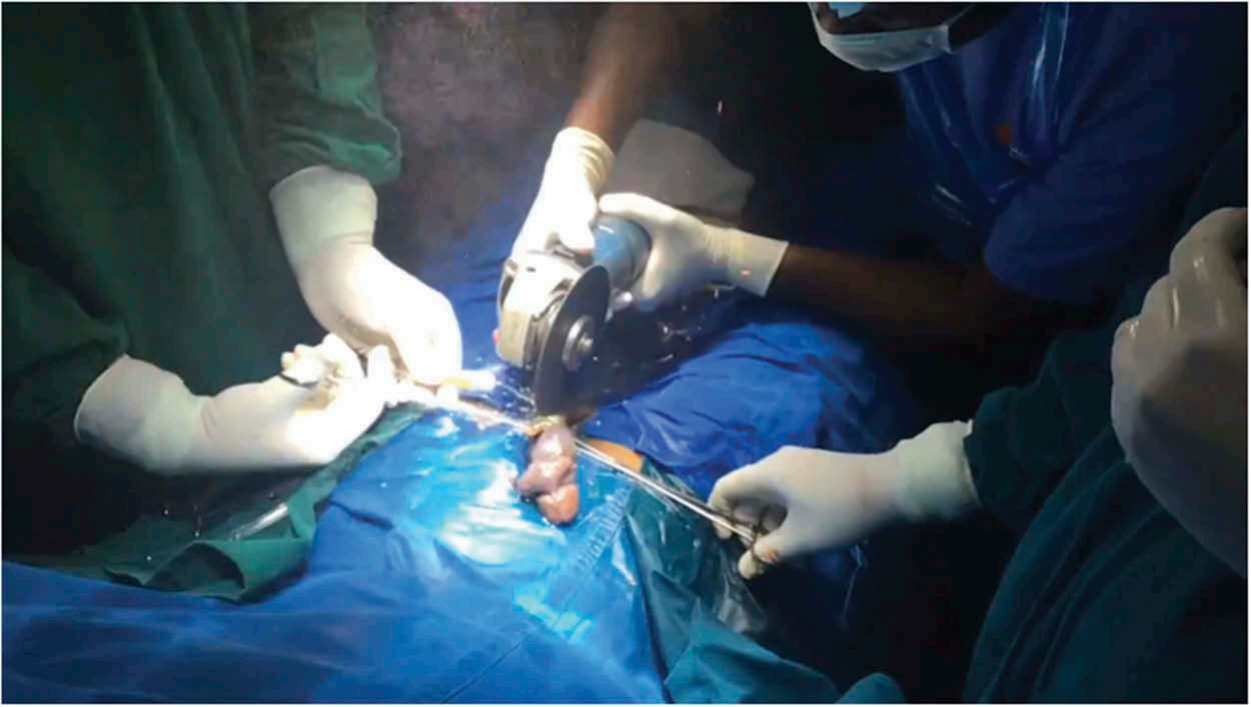
Using angle grinder to cut the foreign body.

**Figure 13.**
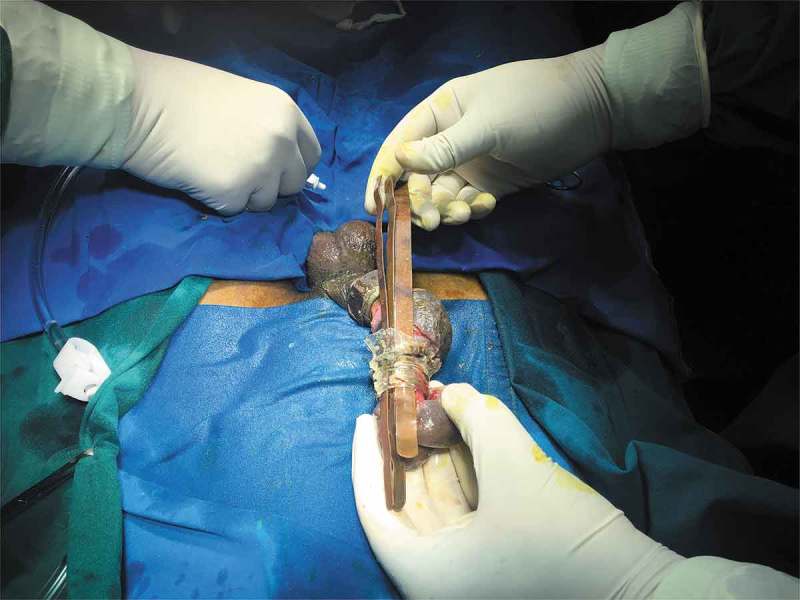
Passing malleable retractors under the foreign body to prevent injury to underlying structures.

**Figure 14.**
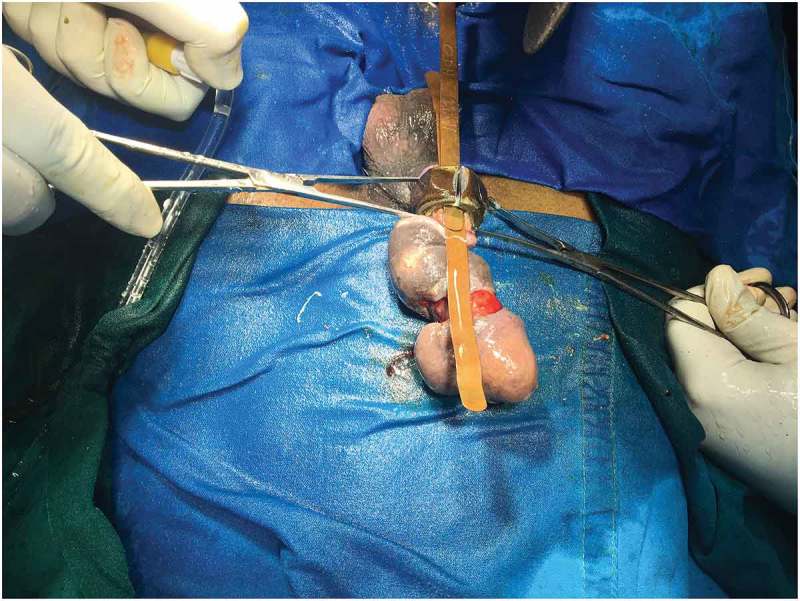
Use of Allis forceps to fix the foreign body for precise cut.

If Level 2 fails, then we immediately go for more powerful tools (Level 3) that can cut through the foreign body quickly. The patient should be properly draped before using Level 3 devices to prevent injury from the sparks produced whilst cutting. As they are very rapid, extreme precision is required to cut the foreign body. Various devices can be used including: orthopaedic power drilling machine, pedal cutter, angle grinder (Figure 12), and reciprocating saw. All these devices, except for the orthopaedic drilling machine, will be available in industrial sites. Thus, if it is suspected that a Level 3 procedure may be required, the availability of these instruments should be ascertained prior to commencement. At our hospital we were lucky to have availability of instruments used by the maintenance department. If the foreign body is a large metallic body, then we immediately go to Level 3 devices to cut the foreign body, e.g., a patient had 4.5-kg (10-lb) iron barbell around the base of his penis causing penile strangulation for which an angle grinder was directly used [18]. There are case reports where firemen were called in for removal of the foreign bodies [19]. An angle grinder was used in two patients in our present study (accompanying video available at: https://drive.google.com/file/d/1PmMfmiaUfe2LG61LJAZ8S2c6NxAZUlXo/view?usp=sharing). Silberstein et al. [20] used an angle grinder to cut a stainless-steel ring strangulating both the penis and scrotum. In our present case series, we also used a pedal cutter to cut the foreign body, which was also used by Sathesh-Kumar et al. [19]. Whilst using Level 3 devices, to prevent accidental injury to the underlying structures, malleable retractors (Figure 13) etc. should be passed underneath the foreign body if possible. While cutting the foreign body, it should be immobilised for stabilisation and precision whilst cutting, and we found large Allis forceps to be suitable (Figure 14). Whilst cutting the foreign body, to prevent thermal injury from the heat generated, continuous water irrigation (Figure 14) or ice should be used.

**Figure 15.**
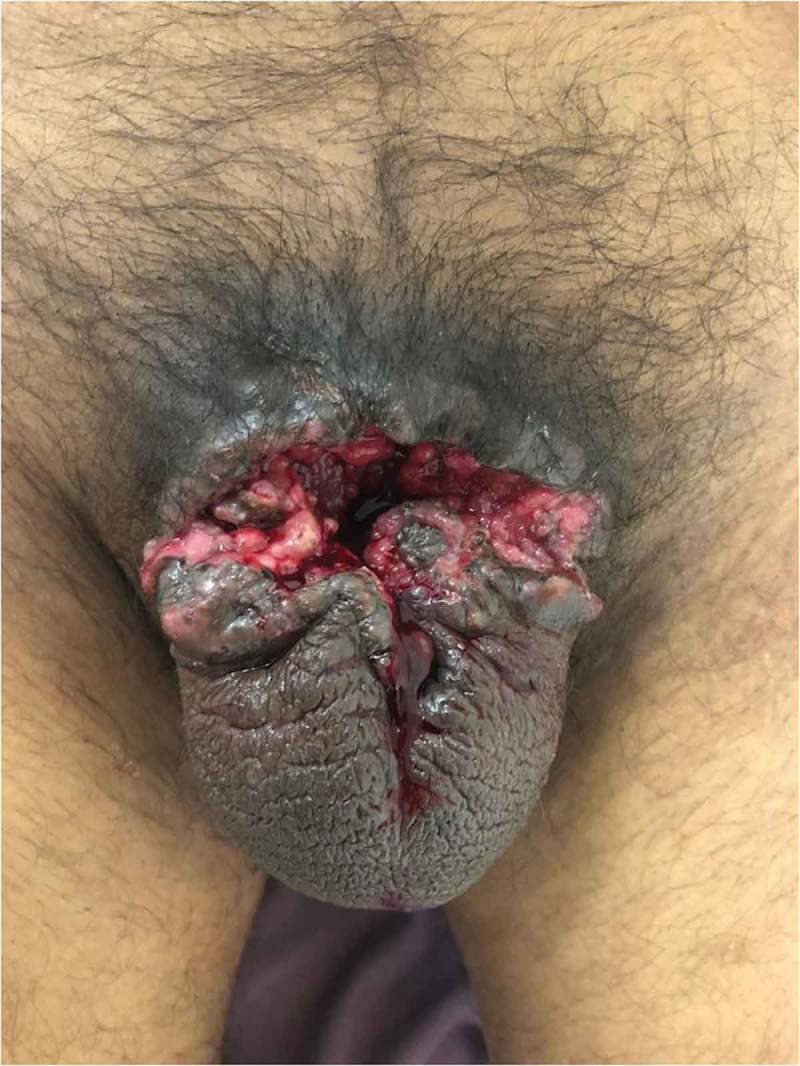
Auto-amputation of penis after penile strangulation with infected base.

In the present study, the duration of penile strangulation varied from 2 h to 2 months, with those presenting late apparently being due to embarrassment. Most of the patients presented with distal penile oedema with a foreign body, and four of the nine had the foreign body in the proximal penile region. Three patients had complications. In three patients, SPC was required as per urethral catheterisation was not possible. One patient, lost to follow-up for 1 year who had his SPC removed by local hospital, later presented back to us with urethro–cutaneous fistula secondary to stricture (Figure 7). The other patient had short segment stricture at the penoscrotal junction and he underwent visual internal urethrotomy with regular uroflowmetry follow-up. One patient presented with auto-amputation of penis (Figure 15), with an infected wound over the detached penile site, so SPC was done as a diversion procedure for healing followed by perineal urethrostomy. One patient developed wound infection for which debridement and skin grafting was done when the wound was healthy.

Table 4 compares the present study with two similar studies.

**Table 4.** Comparison of the present study with other similar studies.

Variable	Present study	Bhat et al. [5]	Shukla et al. [12]
No. of patients	9	8	7
Age, years, range	1–65	6–56	16–80
Distal penile oedema, *n/N*	8/9	8/8	Not assessed
Retention of urine, *n/N*	5/9	4/8	Not assessed
Decreased distal penile sensation, *n/N*	4/9	3/8	Not assessed
Ulceration, *n/N*	4/9	7/8	Not assessed
Patients with a mental disorder, *n/N*	3/9	1/8	None
Duration of strangulation	2 h to 2 months	10 h to 12 days	6 h to 7 days
Grade of injury, *n/N*			
Grade 1	3/9	1/8	1/7
Grade 2	2/9	2/8	5/7
Grade 3	2/9	3/8	1/7
Grade 4	1/9	2/8	–
Grade 5	1/9	–	–
SPC, *n/N*	3/9	2/8	–
Removal techniques	Scissors, string technique with aspiration, bone cutter, orthopaedic saw to cut plaster of Paris, angle grinder, pedal cutter	Scissors, metallic cutter, hammer and chisel, heavy drill, saw	Aspiration with hot compression, scissors, string technique with or without aspiration, dorsal slit
Metallic foreign body, *n/N*	6/9	4/8	7/7
Complications, *n/N*	3/9	1/8	3/7

## Conclusion

Penile strangulation is one of the rare urological emergencies experienced by a urologist. Removal of the foreign body is difficult and there is no universal method for removal as each case differs. So, following our stepwise approach can aid in removal of the foreign body quickly and preserve the penis from fatal outcomes. Urologist should be aware of all the available armamentarium used for the removal of various foreign bodies.

## References

[1] Maruschke M, Seiter H. [Total infarction of the penis caused by entrapment in a plastic bottle]. Urologe A. 2004;43:843–844. [article in German] 15205739 10.1007/s00120-004-0623-5

[2] Ivanovski O, Stankov O, Kuzmanoski M, et al Penile strangulation: two case reports and review of the literature. J Sex Med. 2007;4:1775–1780. 17888068 10.1111/j.1743-6109.2007.00601.x

[3] Noh J, Kang TW, Heo T, et al Penile strangulation treated with the modified string method. Urology. 2004;64:591. 15351614 10.1016/j.urology.2004.04.058

[4] Perabo FG, Steiner G, Albers P, et al Treatment of penile strangulation caused by constricting devices. Urology. 2002;59:137. 10.1016/s0090-4295(01)01485-6 11796305

[5] Bhat AL, Kumar A, Mathur SC, et al Penile strangulation. Br J Urol. 1991;68:618–621. 1773293 10.1111/j.1464-410x.1991.tb15426.x

[6] Markland C, Merrill D Accidental penile gangrene. J Urol. 1972;108:494–495. 4559510 10.1016/s0022-5347(17)60784-5

[7] Snoy FJ, Wagner SA, Woodside JR, et al Management of penile incarceration. Urology. 1984;24:18–20. 10.1016/0090-4295(84)90379-0 6740843

[8] Cassidy DJ, Mador D Genital incarceration: an unusual case report. Can Urol Assoc J. 2010;4:E76–E78. 23293693 10.5489/cuaj.863 PMC3499925

[9] Andalibi MS, Azarfar A, Esmaeeli M, et al Penile hair tourniquet syndrome due to a coil of hair: first report in Iran. Nephro-Urol Mon. 2017;9:e59788.

[10] Acimi S Penile strangulation by hair. Pediatr Surg Int. 2014;30:729–732. 24879557 10.1007/s00383-014-3523-9

[11] Pinggera GM, Pichler R, Rehder P, et al Penile strangulation in a patient with Parkinson’s disease: a case report. Cases J. 2009;2:9379. 20066067 10.1186/1757-1626-2-9379 PMC2804731

[12] Shukla P, Lal S, Shrivastava GP, et al Penile incarceration with encircling metallic objects: a study of successful removal. J Clin Diagn Res. 2014;8:NC01–NC05. 10.7860/JCDR/2014/8755.4447 PMC4129311 25121021

[13] Dong C, Dong Z, Xiong F, et al Successful removal of metal objects causing penile strangulation by a silk winding method. Case Rep Urol. 2013;2013:434397. 24288646 10.1155/2013/434397 PMC3833198

[14] Sawant AS, Patil SR, Kumar V, et al Penile constriction injury: an experience of four cases. Urol Ann. 2016;8:512–515. 28058007 10.4103/0974-7796.192101 PMC5100168

[15] Abd El Salam MA, Gamal A, Elenany H Bone cutting forceps: a safe approach for saving strangulated penis. Case Rep Med. 2016;2016:1274124. 27239200 10.1155/2016/1274124 PMC4864529

[16] Paonam S, Kshetrimayum N, Rana I Penile strangulation by iron metal ring: A novel and effective method of management. Urol Ann. 2017;9:74–76. 28216935 10.4103/0974-7796.198873 PMC5308044

[17] May M, Gunia S, Helke C, et al Penile entrapment in a plastic bottle – a case for using an oscillating splint saw. Int Urol Nephrol. 2006;38:93–95. 16502059 10.1007/s11255-005-8441-2

[18] Santucci RA, Deng D, Carney J Removal of metal penile foreign body with a widely available emergency-medical-services-provided air-driven grinder. Urology. 2004;63:1183–1184. 10.1016/j.urology.2004.01.021 15183986

[19] Sathesh-Kumar T, Hanna-Jumma S, De Zoysa N, et al Genitalia strangulation–fireman to the rescue! Ann R Coll Surg Engl. 2009;91:W15–W16. 10.1308/147870809X400976 PMC2749397 19416581

[20] Silberstein J, Grabowski J, Lakin C, et al Penile constriction devices: case report, review of the literature, and recommendations for extrication. J Sex Med. 2008;5:1747–1757. 18507720 10.1111/j.1743-6109.2008.00848.x

